# HOW CLINICIANS INVOLVE CARE PARTNERS IN THE HEALTH PRIORITIES IDENTIFICATION PROCESS IN DYADS AFFECTED BY DEMENTIA

**DOI:** 10.1093/geroni/igae098.0650

**Published:** 2024-12-31

**Authors:** Joan Monin

**Affiliations:** Yale School of Public Health, New Haven, Connecticut, United States

## Abstract

Care partners play a crucial role in supporting their kin and friends who are living with dementia and multiple chronic conditions (MCCs) in decision making; yet, we know little about how clinicians include care partners in the Health Priorities Identification Process (HPIP) when they are present at clinic visits. We conducted an online survey with 34 clinicians who currently use HPIP with patients living with dementia with MCCs. On average, clinicians reported that at 53% of sessions, a care partner was present. When care partners are present, clinicians reported the extent to which care partners are involved in the following ways: (1) the patient responds to the HPIP questions on their own or with minor support from their care partner (80% of clinicians said sometimes, often, or always); (2) the patient and the care partner work together to respond to the questions (93% said sometimes, often, or always), and (3) the patient almost fully defer to the care partner to answer the questions (70% said sometimes, often, or always). About 87% of clinicians agreed that patients could successfully complete HPIP with a care partner involved; 84% of clinicians said it is easier for them to complete HPIP when a care partner is involved; and 87% said if care partners had more guidance about how to serve as active listener when patients complete HPIP, they believe care partners would be better prepared to make decisions for the patient if the care partner had to serve as a proxy-decision maker.

